# Correlation and agreement between superb micro-vascular imaging and contrast-enhanced ultrasound for assessing radiofrequency ablation treatment of thyroid nodules: a preliminary study

**DOI:** 10.1186/s12880-021-00697-y

**Published:** 2021-11-22

**Authors:** Yu Lan, Nan Li, Qing Song, Ming-bo Zhang, Yu-kun Luo, Yan Zhang

**Affiliations:** 1 Department of Ultrasound, General Hospital of Chinese PLA, 28 Fuxing Road, Haidian District, Beijing, China; 2 School of Medicine, Nankai University, 94 Weijin Road, Nankai District, Tianjin, China; 3 Department of Ultrasound, The People’s Hospital of Liaoning Province, Shenyang, China

**Keywords:** Superb micro-vascular imaging (SMI), Contrast-enhanced ultrasound (CEUS), Radiofrequency ablation (RFA), Volume measurement, Ablation lesion, Agreement, Correlation

## Abstract

**Background:**

To evaluate the correlation and agreement between superb micro-vascular imaging (SMI) mode and the contrast-enhanced ultrasound (CEUS) mode for the ablative completeness and the volumes of ablation lesions to determine the clinical application value of SMI in follow-up after radiofrequency ablation.

**Methods:**

From April 2020 to June 2020, two radiologists used SMI and CEUS mode to measure the volume of the ablation lesion. We use intra-class correlation coefficient (ICC), scatter plots and Bland–Altman plots to evaluate the correlation and agreement of the two techniques. In addition, intra- and inter-observer reliability in volume measurement of ablation lesions with SMI mode was assessed.

**Results:**

SMI mode and CEUS mode have good agreement in the evaluation of ablative completeness. The ICC was 0.876 and 0.928 of reader A and reader B between SMI mode and CEUS mode in terms of ablation lesions volume measurement. There was a strong correlation between the two modes in both reader A and reader B (r_A_ = 0.808; r_B_ = 0.882). The ICC was 0.836 for the inter-observer reliability of SMI technique. The scatter plot showed a good linear relation (r = 0.715). In the Bland–Altman plot, 4.35% (1/23) of the points was outside the 95% limits of agreement. The ICC was 0.965 for the intra-observer reliability of SMI technique, the scatter plot also showed a strong linear correlation (r = 0.965). In the Bland–Altman plot, 8.70% (2/23) of the points was outside the 95% limits of agreement.

**Conclusions:**

SMI and CEUS have good agreement and correlation in the ablation volume measurement. SMI technology is expected to be applied as an alternative to CEUS in the clinical follow-up of ablation lesions.

## Introduction

Thyroid nodule is a common disease and is detected in 19–68% of general population by ultrasound [1]. The vast majority are benign and if malignant, the most common subtype is papillary thyroid carcinoma (PTC). Surgery is the classic treatment for PTC and symptomatic benign nodules. However, it is accompanied by some inevitable complications [2, 3]. In order to avoid the related possible complications, non or minimal invasive thermal ablative option such as laser, radiofrequency, microwaves, ethanol ablation, and high intensity focused ultrasound (HIFU) in the treatment of benign and papillary thyroid microcarcinoma (PTMC) have been diffusing [4–6]. Among them, the safety and efficacy of radiofrequency ablation (RFA) for thyroid nodule has been widely proven [7–9]. Ideally, RFA causes coagulative necrosis of tumor cells. The necrotic tissue is engulfed and absorbed by immune cells, and the ablation lesion gradually shrinks until it disappears [10].

For malignant nodules, whether the lesion has been completely ablated (that is, there is no vascular component in the ablation lesion) is the key to determining whether the treatment is effective. For benign nodules, studies have confirmed that the initial ablation ratio exceeding 80% can better guarantee the ablation efficacy. To ensure the effectiveness of ablation, no matter benign nodules or malignant nodules, the determination of the ablation range is very important for judging the prognosis. However, Color-Doppler US is not sufficiently sensitive to detect small vessels and slow blood flow [11], therefore, evaluating whether the ablated nodule is completely necrotic and the volume of the ablated lesion is still a problem. To overcome these disadvantages of Color-Doppler US, some authors suggested that contrast-enhanced ultrasound (CEUS) can be an ancillary diagnostic tool for detecting the under-ablated portion after RFA [12, 13]. No contrast agent perfusion in the ablation lesion indicated the disappearance of microcirculation in the nodules, which represented the complete ablation of nodules. However, while CEUS is widely used in evaluating the ablation effect [8, 9], clinically it still has disadvantages, such as adverse response and high cost, and patients who are allergic to the components of the contrast agent cannot perform this test.

Superb micro-vascular imaging (SMI) is a new noninvasive ultrasound technique for evaluating microvascular perfusion. The fundamental principle of SMI is to use effective algorithms to distinguish low-speed blood flow signals and tissue motion artifacts, and to separate out effective information to protect small low-speed blood flow signals [14]. Therefore, it can improve the detection sensitivity of microcirculation, and is considered to be an innovation of vascular display in ultrasound. SMI has been used to detect blood flow in thyroid, breast and liver tumors [15–17]. Studies have shown that SMI and CEUS have a high agreement in differentiating benign from malignant thyroid nodules [15]. However, there is little research about SMI for the evaluation of the effectiveness of RFA of thyroid nodules and the volume measurement of ablation lesions.

The aim of our study was to evaluate the correlation and agreement between SMI mode and the CEUS mode for the ablative completeness and the volumes of ablation lesions to determine the clinical application value of SMI in follow-up after RFA, as well as to evaluate inter- and intra-observer agreement in the assessment of volumes of ablation lesions with SMI mode.

## Methods

### Ethical considerations

This study was approved by the Medical Ethics Committee of General Hospital of the Chinese People’s Liberation Army (No. S2019-211-01), and all enrolled people signed informed consent before undergoing CEUS.

### Patients

From April 2020 to June 2020, 20 patients with 23 nodules (20 malignant nodules and 3 benign nodules) who underwent radiofrequency ablation in our hospital and came to our department for follow-up were enrolled in this study.

For the malignant nodules, patients were enrolled in our study if they fulfilled the following criteria: (1) core needle biopsy (CNB) PTMC which is defined as PTC with a maximum diameter of less than 1 cm; (2) without any evidence of nodal or distant metastases, extrathyroidal extension, history of radiation exposure; (3) patients who rejected surgery or have contraindications to surgery. The exclusion criteria were: (1) coagulation dysfunction; (2) CNB confirming PTMC with the aggressive histological type (such as hobnail, poorly differentiated, or tall cell variants); (3) the ablation lesions disappeared during follow-up.

For the benign nodules, the inclusion criteria were as follows: (1) The maximum diameter of the nodule was ≥ 2 cm; (2) the presence of symptoms, such as neck discomfort or difficulty swallowing; (3) affect the appearance; (4) patients who were worried that the lesions will become malignant; (5) patients who rejected surgery or have contraindications to surgery; (6) The lesions confirmed as benign nodules by core needle biopsy (CNB). The exclusion criteria were: (1) coagulation dysfunction; (2) the ultrasound showed malignant features (such as microcalcification, irregular margins, A/T > 1); (3) the ablation lesions disappeared during follow-up.

### Ablation procedure

All patients enrolled in this study underwent routine ultrasound (US) and CEUS before RFA by using Siemens Acuson Sequoia 512 ultrasonic diagnostic instrument (Siemens medical system, Mountain View, CA) with a 15L8W linear array transducer or Philips EPIQ7 ultrasonic diagnostic instrument (Philips Healthcare medical system, Bothell, WA) with a L12-5 linear array transducer or Mindray M9 ultrasonic diagnostic instrument (Mindray medical system, Shenzhen, China) with a L12-4 linear array transducer. Sonographic features of all the nodules including location, composition, echogenicity, shape, margin and echogenic foci (Table 1) were assessed and recorded according to the ACR TI-RADS [18]. All RFA procedures were performed by the same sonographer (Y.K.L.) with clinical experience for 20 years in routine and interventional US.

**Table 1:** Sonographic features of all the nodules

Features	N	Percentage (%)
Location		
Left	9	39.13
Right	14	60.87
Isthmus	0	0
Composition		
Cystic or almost completely cystic	0	0
Spongiform	0	0
Mixed cystic and solid	3	13.04
Solid or almost completely solid	20	86.96
Echogenicity		
Anechoic	0	0
Hyperechoic or isoechoic	3	13.04
Hypoechoic	12	52.17
Very hypoechoic	8	34.78
Shape		
Wider-than-tall	4	17.39
Taller-than-wide	19	82.61
Margin		
Smooth	3	13.04
Ill-defined	3	13.04
Lobulated or irregular	17	73.91
Extra-thyroidal extension	0	0
Echogenic foci		
None or large comet-tail artifacts	4	17.39
Macrocalcifications	0	0
Peripheral (rim) calcifications	0	0
Punctate echogenic foci	19	82.61

Patients lay in the supine position with their necks extended. Skin sterilization was performed and 1% lidocaine was used for local anesthesia at the intended puncture site. The hydro-dissection technique was used with a mixture of 1% lidocaine injected into the anterior capsule space and normal saline injected into the posterior capsule space to protect vital structures (cervical artery, trachea, esophagus, recurrent laryngeal nerve) to prevent thermal injury when the distance between the lesion and the surrounding vital structures was less than 5 mm. Moving-shot ablation technique was used to perform the RFA [19, 20].

### Follow-up examination and image acquisition

From April 2020 to June 2020, in all patients who underwent RFA follow-up in our department, CEUS and SMI examinations were performed unless the ablation lesion disappeared. The ultrasound follow-up process was completed by the same physician (examiner X with > 5 years’ experience with thyroid ultrasound diagnosis) who performed a pre-RFA and both static and dynamic ultrasound images were retained. The two other radiologists (reader A and reader B) read the ultrasound images, in addition, they used the retained dynamic images to re-measure the size of the ablation lesions independently. The reader A and reader B had 5 years and more than 10 years of working experience in thyroid ultrasound examination respectively, and they had conducted over 2000 cases of thyroid ultrasound before this study.

### SMI technique

**Fig. 1:**
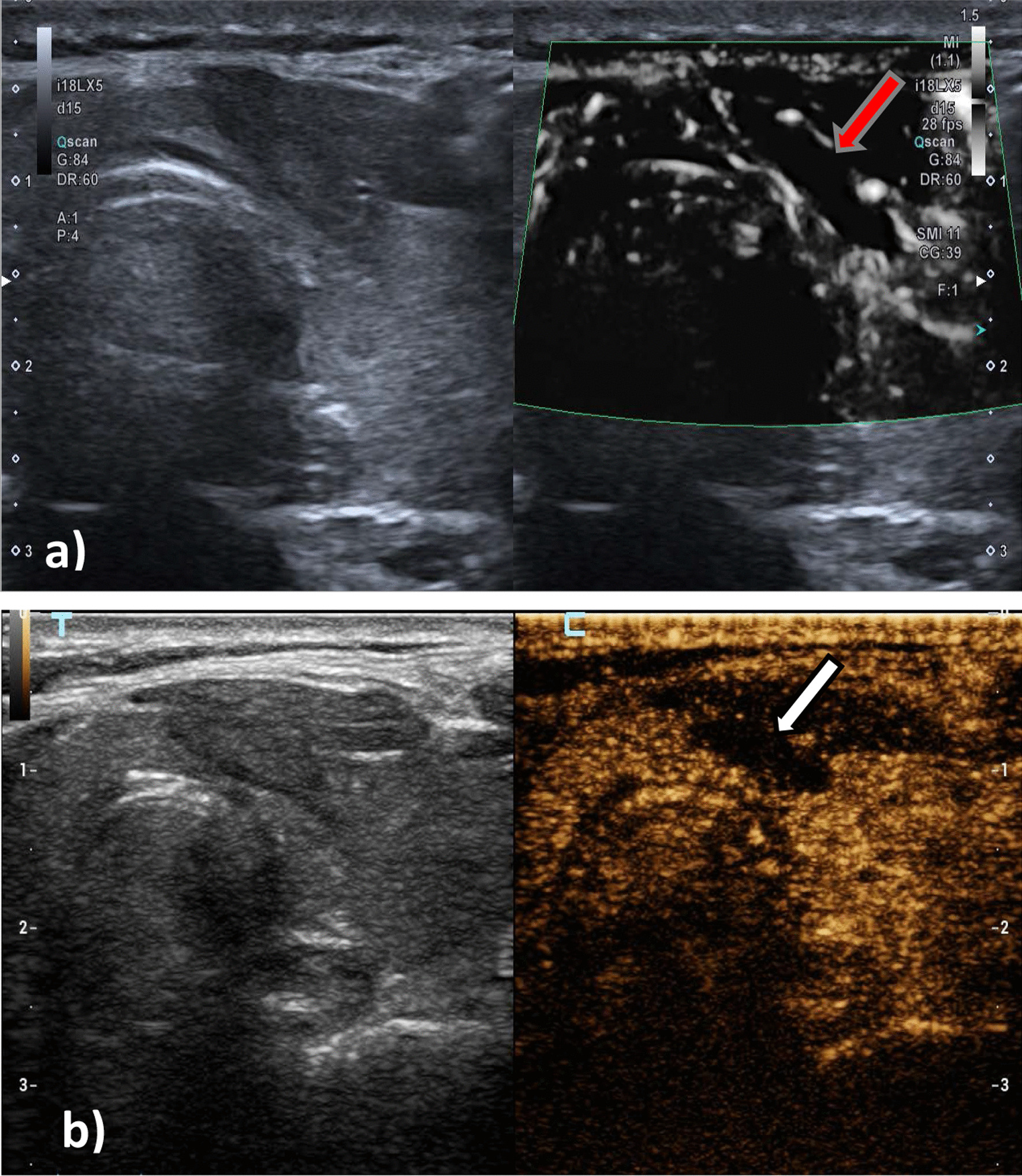
The red arrow in **a** indicates the ablation lesion in SMI mode. No blood flow was detected in the ablation lesion. The white arrow in **b** indicates the ablation lesion in CEUS mode, and no contrast agent perfusion is found in the ablation lesion. The ablation volumes in SMI mode were larger than that in CEUS mode

The SMI was performed by using an APLIO i800 TUS-AI800 ultrasonic diagnostic instrument (Canon Medical Systems, Tokyo, Japan) with an 18L-5 linear array probe. SMI includes both color and monochrome (gray-scale) modes, the gray-scale modes focus more on blood vessels and improve sensitivity by eliminating background information. The sampling frame of SMI covers the entire lesion and a little peripheral thyroid parenchyma (Fig. 1a). In enrolled patients, pulsed Doppler examination was required to verify the authenticity of the blood vessels around the lesion when physicians assessed the ablation lesion in the grayscale mode of SMI. The scale was set at a low-speed scale (1–1.5 cm/s). The areas with no microvascular were measured.

### CEUS technique

CEUS was performed using a Mindray R7 ultrasonic diagnostic instrument (Mindray medical system, Shenzhen, China) with a L12-4 linear array transducer or an APLIO i800 TUS-AI800 ultrasonic diagnostic instrument (Canon Medical Systems, Tokyo, Japan) with an 18L-5 linear array probe. Under the CEUS mode, dynamic dual real-time imaging is applied, with the contrast image on one side and the two-dimensional (2D) gray-scale image on the other side (Fig. 1b). The contrast agent was a second-generation SonoVue (Bracco, Milan, Italy), a phospholipid-coated sulfur hexafluoride gas. After 25 mg of SonoVue was diluted with 5.0 mL of 0.9% saline solution, a microbubble suspension was formed after a full concussion. A bolus of 2.0 mL of contrast agent was injected into the antecubital vein and flushed with 5.0 mL of 0.9% saline solution. Patients were asked to breathe quietly during the dynamic study. The probe was kept in the same position during acquisition, with sagittal or coronal planes being preferred to avoid out-of-plane motion because the lesion had to be visible throughout the respiratory cycle; the ablation lesion should be dynamically observed for 90 s, and dynamic images should be stored.

### The assessment of ablative completeness

In this study, no pathological results of ablation lesions were obtained after RFA. Therefore, in the CEUS mode, perfusion without contrast agent in the ablation lesion was used as the criteria for the determination of complete ablation. In SMI mode, no micro-blood flow was detected in the ablation range, which was considered as complete ablation.

### Volumetric measurement process

Reader A and reader B retrospectively analyzed the retained static and dynamic ultrasound images on the ultrasonic instrument independently. They measured the volumes of ablation lesions in 2D mode, SMI mode and CEUS mode. In order to obtain intra-observer agreement for SMI measurements, a second measurement was performed in SMI mode by reader A after 1 week. Before starting the measurement, in order to obtain the objective measurement results, we specified the measurement method. All measurements were performed by two readers independently. When we measured the ablation lesions, we put the caliper on the outer edge of the nodules. The wide and high diameters were measured on the cross-section, the width is the largest cross diameter on the cross section, and the height is measured perpendicular to the wide diameter. The longest diameter measured on the longitudinal section is the length of the ablation zone. The ellipsoidal formula is used to evaluate the volume of the ablation lesion: length × width × height × π/6.

### Statistical analysis

Wilcoxon’s signed-rank test was used to compare the volumes of the ablation lesions in SMI mode to the volumes in CEUS mode. The spearman coefficient was used for correlation analysis between the volumes of the ablation lesions in SMI mode to the volumes in CEUS. Intra- and inter-observer agreement of volume measurement of the ablation lesions was evaluated by the intra-class correlation coefficient (ICC; mean/single rating, absolute agreement, two-way mixed-effects model). Grading followed the strictest guidelines: < 0.50 = poor; 0.5–0.75 = moderate; 0.75–0.90 = good; > 0.90 = excellent [21]. For the intra-observer agreement, the two measurements of reader A on the same week were analyzed. For inter-observer agreement, the first measurement of reader A was compared to the measurement of reader B. Boxplots, scatter plots and Bland–Altman plots were conducted for visualization. Outliers were defined as the 95% limits of agreement (mean difference ± 1.96 standard deviation). All statistical analyses were performed using SPSS 26.0 (IBM Corp., Chicago, USA) and MedCalc for Windows (version 19.1, MedCalc Software, Ostend, Belgium). *P* < 0.05 was considered statistically significant.

## Results

In this study, 20 patients (23 thyroid nodules including 3 benign lesions and 20 malignant lesions) were enrolled. The mean age of the participants (16 women, 4 men) was 43.39 ± 11.00 years (range: 29–64 years). All the patients were successfully treated with RFA. There were no serious complications such as recurrent laryngeal nerve injury, trachea and esophagus injury, skin burns. The nodules enrolled in this study were determined to be completely ablated in both CEUS mode and SMI mode. The two modes have good agreement in the evaluation of complete ablation. The volumes of ablation lesions measured by reader A and reader B in different modes as were shown in Table 2.

**Table 2:** The volumes of ablation lesions assessed by reader A and reader B in different modes

	Volume of 2D mode	Volume of SMI mode	Volume of CEUS mode
Reader A	0.24 ± 0.19	0.19 ± 0.14	0.14 ± 0.14
Reader B	0.21 ± 0.18	0.17 ± 0.14	0.15 ± 0.17
The second measurement of reader A	0.22 ± 0.15	0.15 ± 0.13	0.13 ± 0.13

### The correlation and agreement of volume measurement of the ablation lesions between in SMI mode and in CEUS mode

**Fig. 2:**
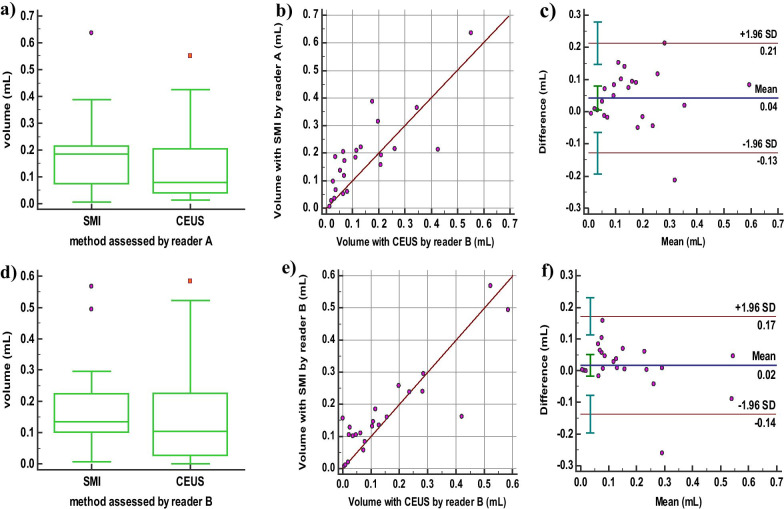
The correlation and agreement of volume measurement of the ablation lesions between in SMI mode and in CEUS mode. **a**, **d** Boxplot of repeated measurement values by reader A and reader B. **b**, **e** Scatter plot of repeated measurement values. **c**, **f** Bland–Altmann plot with the mean of repeated measurement values on the x-axes, and the difference of the measurements and their mean on the y-axis

The volumes of ablation lesion measured by SMI and CEUS for both reader A and B were statistically different (P_A_ = 0.016; P_B_ = 0.026) (Fig. 2a, d). The ICC was 0.876 which was defined as “good” of reader A between SMI mode and CEUS mode (95% confidence [CI] 0.683–0.949, *P* = 0.000) as well as the ICC was 0.928 which was defined as “excellent” of reader B (95% CI 0.833–0.969, *P* = 0.000). The ICC was 0.802 which was defined as “good” of reader A between 2D mode and CEUS mode (95% confidence [CI] 0.287–2.930, *P* = 0.000) while the ICC was 0.704 which was defined as “moderate” of reader B (95% CI 0.324–0.873, *P* = 0.000). It indicates that the volume measurement agreement between SMI mode and CEUS mode is higher than that between 2D mode and CEUS mode for both readers. The scatter plot revealed that the measured volumes of reader A and B were linearly correlated in SMI mode and CEUS mode. (r_A_ = 0.808, *P* = 0.000; r_B_ = 0.882, *P* = 0.000) (Fig. 2b, e). In the Bland–Altman plot, 4.35% (1/23) of the points was outside the 95% limits of agreement (Fig. 2c, f).

### The inter-observer reliability of ablated volume measurement with SMI mode

**Fig. 3:**
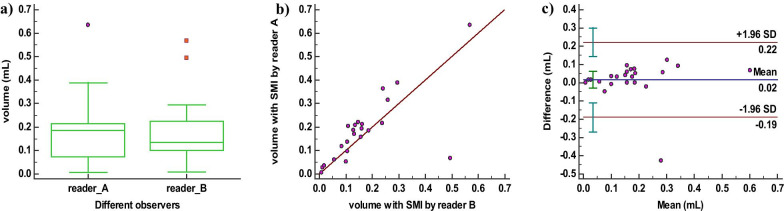
Inter-observer reliability for SMI mode. **a** Boxplot of repeated measurement values by reader A. **b** Scatter plot of repeated measurement values. **c** Bland–Altmann plot with the mean of repeated measurement values on the x-axes, and the difference of the measurements and their mean on the y-axis

Comparison of the volumes assessed by the two readers revealed a mean volume of 0.19 mL for reader A and 0.17 mL for reader B, respectively. The Wilcoxon test revealed statistical differences (*P* = 0.011) (Fig. 3a) for volumes assessed by the two readers, however, the ICC was 0.836 which was “good” and highly significant (95% CI 0.616–0.930, *P* = 0.000). The scatter plot showed a good linear relation (r = 0.715) (Fig. 3b). In the Bland–Altman plot, 4.35% (1/23) of the points was outside the 95% limits of agreement (Fig. 3c).

### The intra-observer reliability of ablated volume measurement with SMI mode

**Fig. 4:**
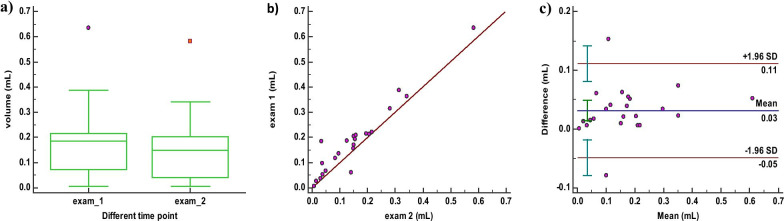
Intra-observer reliability for SMI mode. **a** Boxplot of repeated measurement values by reader A for the first examination and the second examination. **b** Scatter plot of repeated measurement values. **c** Bland–Altmann plot with the mean of repeated measurement values on the x-axes, and the difference of the measurements and their mean on the y-axis

The mean volumes of two measurements repeated by reader A during 1 week were 0.19 mL and 0.15 mL, respectively. The Wilcoxon test revealed statistical differences (*P* = 0.011) for volumes assessed for two measurement by reader A (*P* = 0.000) (Fig. 4a). The ICC was 0.965 which was “excellent” and highly significant (95% CI 0.841–0.988, *P* = 0.000), the scatter plot (Fig. 4b) showed a strong linear correlation (r = 0.965). In the Bland–Altman plot, 8.70% (2/23) of the points outside the 95% limits of agreement (Fig. 4c).

## Discussion

In the present study, we attempted to quantitate the correlation and agreement of the volume measurement of ablation lesions with SMI mode and with CEUS mode. Our data demonstrated that different observers used SMI technology to evaluate the ablative completeness and the volume of ablation lesions showed a linear correlation (r > 0.7) with the results evaluated by CEUS technology and had a good agreement (ICC > 0.8). Our study also demonstrated that the evaluation of the volume of ablation lesions in the SMI mode has a high intra-observer and intra-observer consistency, but the intra-observer agreement is higher than inter-observer.

These data have clinical implications on follow-up microcirculation examinations that are conducted to estimate the ablative completeness, whether there are benign nodules regeneration and recurrence of the malignant nodules, and changes in the volume of ablation [22]. If the microvascular is detected in or peripheral the ablation lesion or the volume of the lesion does not decrease continuously for the malignant lesions, the fine-needle aspiration (FNA) biopsy or core needle biopsy (CNB) should be performed to confirm whether there is residual or recurrence [23]. However, for benign nodules, micro-blood flow is detected in the ablation lesions, which may be due to regrowth of the nodules. In this case, secondary ablation or surgical treatment should be considered if the patient has compression symptoms.

To prevent residual and recurrence, the RFA extent completely covers or exceeds the primary lesion. When all the ablation lesions become hyperechoic which is the tissue vaporized by heat, the ablation stops. The hyperechoic areas represent the area of tissue necrosis. However, over time, the echo of these necrotic tissues changes. In 2D gray-scale images, it is difficult to distinguish the ablation lesion from the peripheral thyroid parenchyma, and it is impossible to accurately determine whether there is residual and recurrence [12]. Our study also confirmed that the 2D assessment of ablative completeness and the volume of ablation lesion has low accuracy.

Previous studies have reported that CEUS can be used as a tool for the evaluation and follow-up of complete ablation [24]. There is no blood supply to the ablated necrotic tissue, and the lesions successfully ablated in CEUS mode show no enhancement zone without contrast agent perfusion, which has high sensitivity and accuracy [13, 25, 26].

Although CEUS is the preferred method of follow-up for ablation lesions, it is invasive and expensive, requiring the use of contrast agents. SMI has been applied as a relatively new noninvasive blood flow imaging mode. Compared with conventional US, SMI can detect low-velocity blood flow more sensitively and display more microvascular information [22, 27]. It includes grayscale and color modes. Grayscale mode highlights blood perfusion by subtracting the information of 2D tissue. Both color mode and gray scale mode can better detect the micro-blood flow and low-speed flow without contrast agent, and can truly reflect the perfusion status in and periphery lesion. Machado et al. found SMI could provide more detailed information about micro-blood flow within the thyroid nodule and show the branching structure of blood vessels [27]. In our study, the grayscale mode of SMI was used to evaluate the completeness of the ablation lesion, and the image showed a filling defect of the blood flow. In the comparison of SMI and CEUS, a good agreement is obtained. In addition, the ablation volumes measured using the SMI mode have a good agreement and strong correlation with CEUS for both reader A and reader B.

Our study found that the volume of ablated lesions measured in 2D mode was greater than the volume measured in SMI mode and CEUS mode. This is consistent with the results of a study reported by Liu et al. [22], evaluating post-percutaneous laser ablation (PLA) lesions. This may be due to the appearance of a transition area between the ablation zone and the surrounding normal thyroid parenchyma. This area is composed of a large amount of granulation tissue, fibroblasts, inflammatory cells and blood capillary hyperplasia of inflammation in which contrast agent can be perfusion [28–30], but in 2D mode, it is different from that of normal thyroid tissue to be mistaken for ablation zone. In addition, we found that the ablation volumes measured in SMI mode were larger than that measured in CEUS mode (Fig. 1). This may be due to the spillover of the contrast agent resulting in a reduction in the unenhanced extent of the true ablation area. Therefore, the dose of contrast agent should be strictly controlled during CEUS, so as to improve the accuracy of contrast measurement of ablation volume.

To our knowledge, no study has reported both intra-observer and inter-observer agreement in the SMI measurements of ablation lesions. The fact that the intra-observer and inter-observer agreement in the SMI measurements of ablation lesions are good, in addition, the intra-observer agreement (ICC = 0.965) is higher than the inter-observer agreement (ICC = 0.836). It implies that the volume measurement of ablation lesion in SMI mode would be more reliable if they are conducted by a same examiner.

There are some limitations in this study. First, the patient population was inhomogeneous, with nodules that were benign or malignant and the follow-up time of patients ranging from 1 to 23 months was different. Second, there were no pathological results available in this study, which is the gold standard for evaluating the ablative completeness. Finally, the number of patients enrolled in this study was small. Therefore, studies requiring long-term follow-up with large samples are expected.

## Conclusions

In conclusion, SMI is an alternative imaging modality to CEUS for visualizing the ablation zone. In addition, we found considerable intra- and inter-observer reliability in volume measurement of ablation lesions with SMI mode. Therefore, SMI technology is expected to be applied in the clinical follow-up of ablation lesions.

## Data Availability

Not applicable.

## Abbreviations

SMI: Micro-vascular imaging
CEUS: Contrast-enhanced ultrasound
RFA: Radiofrequency ablation
ICC: Intra-class correlation coefficient
PTC: Papillary thyroid carcinoma
PTMC: Papillary thyroid microcarcinoma
VRR: Volume reduction rate
